# The evolutionary characteristics and structural biology of Gallus toll‐like receptor 21

**DOI:** 10.1002/jmr.2696

**Published:** 2017-12-27

**Authors:** Hongping Wu, Hai Wang, Wuqi Jiang, Zhengxing Lian

**Affiliations:** ^1^ Beijing Key Laboratory of Animal Genetic Improvement China Agricultural University No.2 Yuanmingyuan West Rd, Haidian Beijing 100194 China; ^2^ College of Animal Science & Veterinary Medicine Shenyang Agricultural University No.120 Dongling Rd, Shenhe Shenyang 110866 China

**Keywords:** docking, Gallus toll‐like receptor 21 (gTLR21), homology model, leucine‐rich repeat (LRR) motif, toll‐IL‐1 receptor (TIR) domain

## Abstract

Toll‐like receptors (TLRs) are an important part of the innate immune system, acting as a first line of defense against many invading pathogens. The ligand known to bind Gallus toll‐like receptor 21 (gTLR21) is the unmethylated cytosine phosphate guanine dideoxy nucleotide motif; however, the evolutionary characteristics and structural biology of gTLR21 are poorly elaborated. Our results suggest that gTLR21 is phylogenetically and evolutionarily related to the TLR11 family and is perhaps a close ortholog of the Mus TLR13. Structural biology of homology modeling of the gTLR21 ectodomain structure suggests that it has no Z‐loop like that seen in Mus TLR9. The cytosolic toll‐IL‐1 receptor region of gTLR21 contains a central 4‐stranded parallel β‐sheet (βA‐βD) surrounded by 5 α‐helices (αA‐αE) on both sides, a highly conserved structure also seen in other TLRs. Molecular docking analysis reveals that the gTLR21 ectodomain has the potential to distinguish between different ligands. Homodimer analysis results also suggest that Phe842 and Pro844 of the BB loop and Cys876 of the αC helix in gTLR21 are conserved in other cytosolic toll‐IL‐1 receptor domains of other TLRs and may contribute to the docking of homodimers. Our study on the evolutionary characteristics and structural biology of gTLR21 reveals that the molecule may have a broader role to play in innate immune system; however, further experimental validation is required to confirm our findings.

**Abbreviations**
CpG‐DNA: cytosine phosphate guanine dideoxy nucleotide motif
ECD: ectodomain
gTLR21: *Gallus* TLR21
IL‐1: interleukin‐1
IRF7: Interferon regulatory factor 7
LRR: leucine‐rich repeat
LRRhs: highly conserved leucine‐rich repeat segments
LRRvs: variable leucine‐rich repeat segment
MyD88: Myeloid differentiation primary response gene 88
PAMPs: pathogen‐associated molecular patterns
ssRNA: single‐stranded RNA
TIR: cytosolic Toll‐IL‐1 receptor region
TLR: Toll‐like receptors

## 1 INTRODUCTION

Toll‐like receptors (TLRs), which are fundamental sensor molecules in the innate immune system, are membrane‐bound receptors located on the cell surface or in endocytic compartments and can recognize a wide range of pathogen‐associated molecular patterns (PAMPs).^1^ All of TLRs usually contain a series of leucine‐rich repeat (LRR) motifs, a transmembrane region, and a cytosolic Toll‐IL‐1 receptor (TIR) domain. The LRR motifs in the ectodomains (ECDs) of different TLRs can recognize different PAMPs as “ligands,” including molecules such as lipids, lipoproteins, proteins, and nucleic acids derived from a wide range of microbes such as bacteria, viruses, parasites, and fungi.^2^ Meanwhile, the TIR domains of TLRs can also trigger downstream signaling pathways to induce the release and production of inflammatory cytokines, type I interferon, and other immune factors. These reactions not only mediate defensive responses such as inflammation but also activate antigen‐specific adaptive immune responses.^3^

So far, 39 types of TLR family members have been identified in organisms via the LRRfinder database (http://www.lrrfinder.com/index.php). A total of 13 TLRs (TLRs 1‐13) have been identified in mammals, mainly in mice and humans; however, the expression patterns of TLRs 10‐13 appear to be species specific.^4^ TLRs 1‐9 are conserved in humans and mice, and their immunological functions have received much attention and in‐depth study. On the basis of their localization, these TLRs are largely divided into 2 subfamilies—cell‐surface TLRs and intracellular TLRs.

Cell‐surface TLRs include TLR1, TLR2, TLR4, TLR5, TLR6, and TLR10, which mainly recognize microbial membrane components such as lipids, lipoproteins, and proteins. TLR2 can heterodimerize with TLR1 or TLR6 and recognize lipoproteins and peptidoglycans from Gram‐positive bacteria. TLR4 and TLR5 recognize the lipopolysaccharides of Gram‐negative bacteria and bacterial flagellin, respectively. TLR10 is a pseudogene in mice, but the human TLR10 can collaborate with TLR2 to recognize ligands from Listeria and is involved in sensing influenza A viral infections.^5^

Intracellular TLRs (including TLR3, TLR7, TLR8, TLR9, TLR11, TLR12, and TLR13) are localized in the endosomal compartments and recognize nucleic acids originating from bacteria and viruses, as well as self‐nucleic acids in disease conditions such as autoimmunity.^1^ TLR3 has been found to recognize viral double‐stranded RNA.^6^ Murine TLR7 and human TLR8 predominantly function in detecting GU‐rich single‐stranded RNA (ssRNA) from viruses.^7^ TLR9 and TLR13 recognize unmethylated cytosine phosphate guanine dideoxy nucleotide (CpG‐DNA) motifs and bacterial 23S ribosomal RNA, respectively.^8^, ^9^ Interestingly, TLR11 responds to flagellin, much like TLR5.^10^ In a recent study, TLR12 was found to be highly similar to TLR11 and functions in recognizing profilin from *Toxoplasma gondii*.^11^

Several types of TLRs have been identified in other vertebrates, including fish and birds. Our current knowledge of *Gallus* TLRs (gTLRs) has been greatly advanced by the assembly of the genome sequence of the chicken (Gallus gallus). Previous studies have demonstrated the presence of 10 TLRs in *Gallus*. The gTLRs 3, 4, 5, and 7 are close orthologs of the corresponding TLRs found in other vertebrates and have similar immunological functions.^4^ In *Gallus*, the mammalian TLRs 1, 6, and 10 are replaced by TLR1La and TLR1Lb from an evolutionary point of view.^12^ The duplicated genes (TLRs 2a and 2b) of *Gallus* are both orthologs of the single TLR2 found in mammals. *Gallus* TLR21 is an ortholog of the TLR21 in fish and amphibians.^13^ It appears that TLR15 is unique to birds and some reptilian species. TLR15 responds to in *Salmonella enterica* infections and is reported to have a unique auto‐activation mechanism.^14^ The avian TLR21 is an ortholog of the TLR21 proteins from teleost and amphibian species, and clusters into the TLR11 subfamily. Surprisingly, gTLR21 has been shown to localize to the endoplasmic reticulum in cells transfected with the gTLR21 gene and can recognize unmethylated CpG‐DNA; therefore, it functions much like TLR9 in mice.^15^, ^16^, ^17^

To date, no 3D structure of gTLRs has been obtained by X‐ray crystallography with a high level of confidence. Furthermore, no experimental data on the detailed molecular structure of gTLR21 complexes are as yet available. Computational methods may reveal information that is not easy to obtain by experimental means, and therefore, it is necessary for us to use computational methods to facilitate biological research. In this study, we use the computational methods to investigate the genetic evolution of gTLR21 and predict different binding patterns that the gTLR21 protein may have with potential ligands. The purpose of our study is to improve our understanding of gTLR21 protein biophysics; the techniques we describe here may be used as tools for identifying potential binding locations and catching sight of exploring novel competitive inhibitors, biosensors, network components, and in vaccine development.

## 2 MATERIALS AND METHODS

### 2.1 Phylogenetic analysis

In this study, sequences of all full‐length TLR proteins (not including the protein whose structure is being predicted) derived from known vertebrate species belonging to mammals, reptiles, avians, and teleosts were downloaded from the Uniprot database (http://www.uniprot.org/). First, aligned sequences for the TLR proteins were generated with MAFFT (L‐INS‐i).^18^ Following this, a phylogenetic tree was constructed using the neighbor‐joining method with the JTT + I + G substitution model developed^19^ by Prottest3.4 and bootstrap sampling was performed 1000 times. The display, annotation, and management of phylogenetic trees was performed in iTOLv3.^20^

### 2.2 Analysis of residue conservation and secondary structural elements

The alignment results obtained in the first step were submitted to the ConSurf algorithm for the evaluation of evolutionarily conserved amino acid residue positions.^21^ The conservation scale ranged from 1 to 9 grades representing different degrees of conservation at the residue positions in gTLR21. The secondary structures of TLR proteins are distinguished through LRRfinder.^22^

### 2.3 Template searching, homology modeling, and interfacing analysis for the LRR region of gTLR21 with differential potential ligands

Because of low sequence identity between the target and template proteins (<40%), we chose to use the best available templates for multiple homology modeling for the LRR region of gTLR21. For this, human TLR3 (PDB ID: 1ziw), monkey TLR7 (PDB ID: 5gmh), human TLR8 (PDB ID: 3wn4), horse TLR9 (PDB ID: 3wpc), and mouse TLR13(PDB ID: 4z0c) were chosen, as these proteins have structures available with 2.1, 2.2, 1.81, 1.6, and 2.3 Å resolution, respectively. A total of 100 candidate models for the target protein were constructed and optimized by Modeller9.18.^23^ To acquire highly reliable model structures, the stereochemical quality and distribution of residual energy for the candidate structures were calculated by SAVES (http://services.mbi.ucla.edu/SAVES)^24^, ^25^, ^26^ and ProSA‐web (https://prosa.services.came.sbg.ac.at/prosa.php),^27^ respectively.

The model of gTLR21 ECD obtained was submitted to the Metapocket2.0 server (http://projects.biotec.tu-dresden.de/metapocket/index.php)^28^ for predicting potential ligand binding sites. CpG‐DNA and ssRNA ligands were separated from the proteins with PDB IDs 3wpc and 4z0c, respectively, and saved in the pdb format using Modeller9.18. Ligands and LRR models are submitted to HDOCK server (http://hdock.phys.hust.edu.cn/),^29^ respectively.

### 2.4 Template searching, homology modeling, and homodimer prediction for TIR region of gTLR21

The TIR regions of human TLR6 (PDB ID: 4om7) and TLR10 (PDB ID: 2j67) for which 2.2 Å resolution structures are available were used as templates for modeling the TIR region of gTLR21 in Modeller9.18. The final target model was evaluated as described in the previous section.

The final structure of the gTLR21 TIR domain as a monomer was submitted to the GalaxyGemini server for exploring protein‐protein interactions^30^ (http://galaxy.seoklab.org/cgi-bin/submit.cgi?type=GEMINI). In our study, all representational structures were displayed with PyMOL.^31^

## 3 RESULTS AND DISCUSSION

### 3.1 gTLR21 belongs to the TLR11 family and is clearly an ortholog to *Mus* TLR13

**Figure 1:**
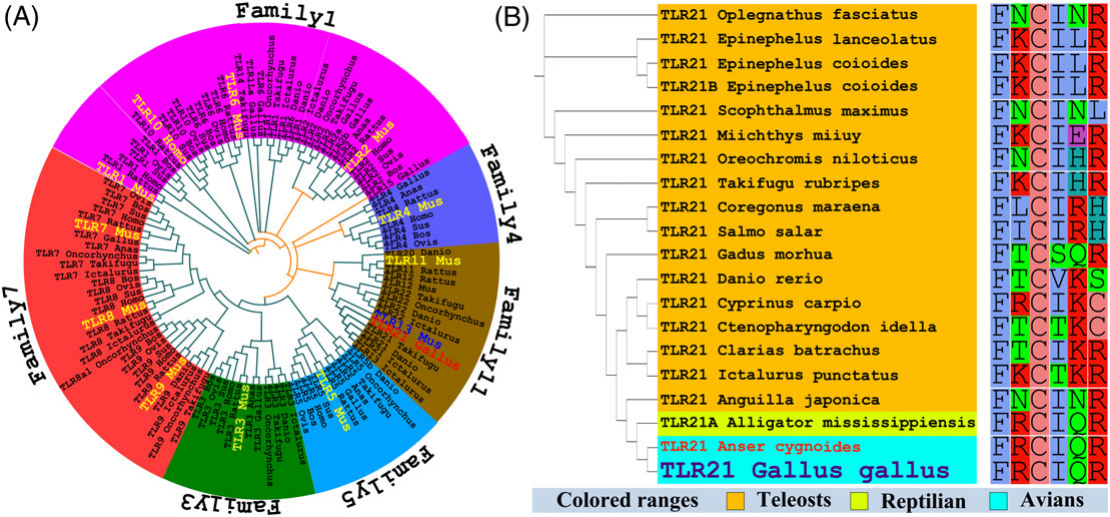
Phylogenetic trees of Gallus TLR21 (gTLR21) and other vertebrate TLRs based on the neighbor‐joining method. A, This phylogenetic analysis shows that TLRs can be divided into 6 subfamilies in vertebrates and that gTLR21 is assigned to Family11. The each subfamily has own color. B, gTLR21 is highly orthologous to Anser cygnoides, and the TLR21 subfamily is conserved in different species

We used known sequences of full‐length TLR proteins from vertebrates to construct phylogenetic relationships based on the neighbor‐joining method (Figure 1A). In the phylogenetic tree obtained, all TLR protein sequences were divided into 6 families. Obviously, TLR21 was found to cluster within the TLR11 family and was clearly an ortholog of *Mus* TLR13. This phylogenetic analysis was consistent with the earlier findings.^32^

Besides building phylogenetic relationship tree, we also analyze the conserved amino acid residues in the complete TLR21 protein sequences of reptilian, avian, and teleost species (Figure 1B). The gTLR21 owns equally high amino acid identities to Anser TLR21, it is possible that TLR21 among avians are highly orthologue and the avian TLR21s are closely related to reptilian. Furthermore, TLR21 is not found in humans or other mammals. Although TLR21 appears to have been lost in most vertebrates, it is particularly present in a minority of vertebrates like some reptilians, avians, and several fish. These data indicate that the evolution of TLR21 is according to the phylogeny of species and probably subjected to the species‐specific constraints.^13^, ^33^

The TLR11 family includes 2 TLR subfamilies—TLRs 11‐13 and TLRs 20‐22; these members of the TLR11 family are probably derived from the TLR1 lineage.^33^, ^34^ The results of our phylogenetic relationship analysis indicate that the TLR11 family can be split into 3 subfamilies (including TLRs 11, 13, and 22). Consistent with previous studies, the clade with the TLR subfamilies 4, 11, and 15 subsequently cluster into the TLR1 family, along with the TLR2 subfamilies.^34^, ^35^ The results of the phylogenetic analysis of TLR21 indicate gTLR21 is highly orthologous to other avian TLR21s; TLR21 proteins are not unique to birds, but are also wide spread in reptilian and teleost species.^13^ The analysis of conserved amino acid residues using complete sequences of TLR21 proteins from different species indicates that the sequences share high identity with each other. This result further provides support for the phylogenetic relationships between the avian, reptilian, and teleost TLR21 proteins.

### 3.2 Structural analysis of the ECD and intracellular domains of gTLR21

**Figure 2:**
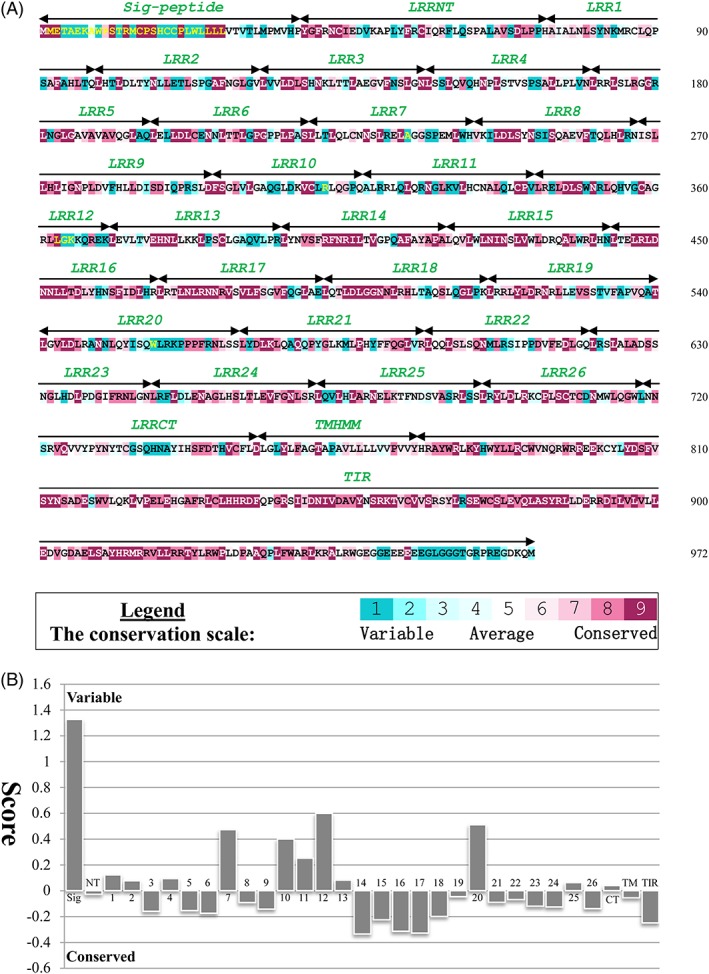
Evolutionarily conserved amino acid positions and mean evolutionary conservation rates for each TLR21 motif. A, The conservation scale ranges from 1 to 9; each glade has a different color, and glades 1 and 9 represent the most variable and most conserved residue positions, respectively. The sequence of Gallus TLR21 (gTLR21) is used to indicate the 9‐color conservation grade system. The signal peptide, each predicted LRR modules of the ectodomain (ECD), transmembrane region (TM), and intracellular domain (TIR) for gTLR21 are labeled. B, The different modules of the gTLR21 protein are represented by their identifying numbers. The lowest score represents the most conserved position in the protein

TLR21 proteins are generally 965 to 986 amino acids in length, and each TLR21 protein has a signal peptide, an ECD (which includes an LRRNT, 26 LRR motifs, and an LRRCT sequence), a transmembrane region, and an intracellular TIR domain, as indicated by motif prediction analysis. Our ConSurf results show that different modules in the TLR21 subfamily have different conserved residue positions. Most of the variable residues are located on the signal peptide, whereas most of the conserved amino acid positions are considered as spanning from the LRRNT to LRRCT in the ECD; similar patterns were also found in the TIR domain (Figure 2A). Meanwhile, we also statistics the average evolutionary conservation score about each motif (Figure 2B). It was observed that LRRs 7, 10‐13, and 20 have relatively fast evolution rates. On the other hand, LRRs 14‐18 have relatively higher conservation scores than other LRR motifs, implying that perhaps they play a role in protein‐protein interfacing for dimerization or in binding to other members of the TLR family.

**Figure 3:**
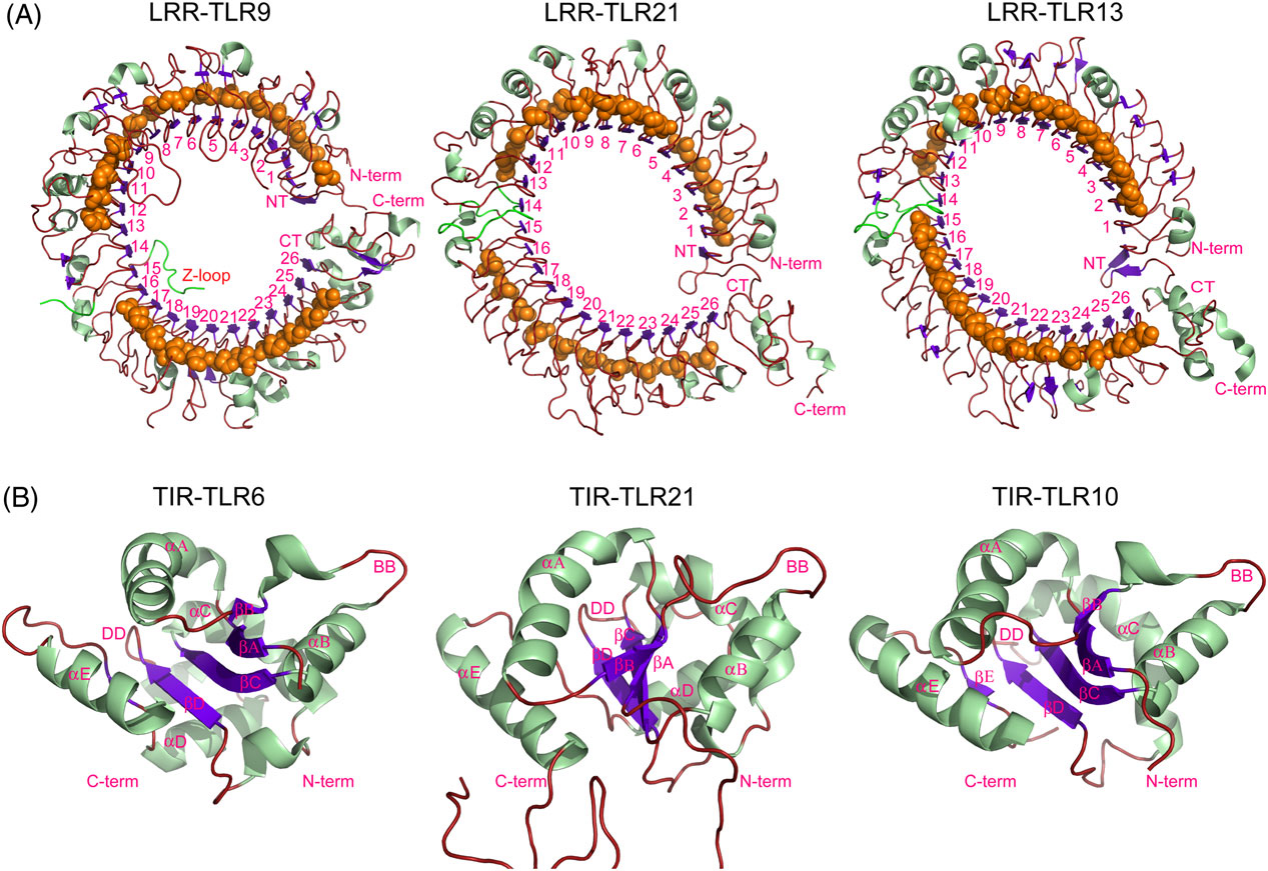
Structural comparison of the ectodomains (ECD) and intracellular domains (TIRs) of different toll‐like receptors (TLRs). A, No Z‐loop was detected between the LRR14 and LRR15 modules in the middle of the gTLR21 ECD; this is markedly different from the crystal structure of the human TLR9 ECD (PDB code: 3wpc), but is similar to the TLR13 ECD (PDB code: 4z0c). B, The overall conformation of the TIR domain of gTLR21 has a typical TIR domain fold like other TLRs (PDB codes: 4om7 and 2j67, respectively)

The structure of gTLR21 ECD was modeled and optimized with Modeller9.18. The scores for the stereochemical quality of the candidate structures calculated by SAVES show that the derived structures of the gTLR21 ECD are reasonable (Tables **S1**A, S2A, and S3A). Simultaneously, the score of target model is −6.02 in ProSA‐web, which indicates that its distribution of residual energies is also acceptable (Figure S4A). The number of LRR motifs in TLR21 is consistent with those observed in *Mus* TLR13 in the family11 and *Mus* TLR9 in the family7. Each LRR motif comprises approximately 22 to 33 residues, except for the LRRCT and LRRNT sequences in the gTLR21 ECD. The LRR motifs can be classified into 2 types—highly conserved segments (LRRhs) and variable segments (LRRvs) in general. The sequence LxxLxLxxN/C(x)xL is an LRRhs‐class model sequence, where “L” represents Leu, Ile, Val, or Phe; “N” represents Asp, Thr, Ser, or Cys; and “x” represents any amino acid.^32^ The LRRhs of gTLR21 are similar to conserved LRR subtypes and can form β‐sheets packing the concave surface of the gTLR21 ECD, the remaining “irregular” LRR motifs bear variable similarities to different subtypes and form a convex surface structure (Figure 3A). It is obvious that gTLR21 can form a noncanonical horseshoe‐shaped structure where the N‐ and C‐terminal ends of protein extensively interact with each other; this is similar to the “closure” of an oval‐shaped structure formed by *Mus* TLR13.^9^ When comparing the LRR modules of gTLR21, *Mus* TLR13, and *Mus* TLR9, an interesting observation was that the 14th LRR motif in gTLR21 lacks the long insertion known as a “Z‐loop” (Figure 3A). The Z‐loop is necessary for TLR9 dimerization and is involved in recognizing ligands for TLRs 7 and 8.^36^ These results indicate that gTLR21 may have its own unique patterns for the recognition of ligands.

In this study, we have demonstrated that TLR21 is more likely to form an oval‐shaped structure like TLR13, rather than a canonical horseshoe‐shaped structure (Figure 3A). We could not detect the presence of a Z‐loop between LRRs 14 and 15 through our model‐building of the gTLR21 ECD. As previously reported, the Z‐loop is considered indispensable for members of the TLR7 subfamily. TLR9 with the uncleaved Z‐loop is unable to form a dimer,^8^ and the Z‐loop is also required for ssRNA recognition by TLRs 7 and 8.^7^, ^37^

The structure of the gTLR21 TIR domain was also modeled and optimized with Modeller9.18 in this study. The scores for the stereochemical quality of the candidate structure calculated by SAVES as previously mentioned show that the structure obtained is reasonable (Figures **S1**B, S2B, and S3B). Simultaneously, the target model has a score of −4.07 in ProSA‐web, which indicates that the distribution of residual energies of the model is also acceptable (Figure S4B). Usually, approximately 125 to 200 amino acid residues in the TIR domain are necessary for protein‐protein interaction. Conserved residue position statistics indicate that amino acids 778‐943 form a highly conserved structure in the cytoplasmic region of gTLR21 (Figure 2A). Our target model suggests that gTLR21 has a typical TIR domain fold with a central 4‐stranded parallel β‐sheet (βA‐βD) surrounded by a total of 5 α‐helices (αA‐αE) on both sides (Figure 3B). The predicted TIR model has 5 extended loops such as BB and DD loops, which are similar to those in human TLR6. The BB loop is involved in interactions with several TIR domains.^3^, ^38^

We have identified the overall conformation of the gTLR21 TIR domain and found that it is similar to others in the TLR family. An important part of the structural stability of the TIR domain is likely to be provided by a 4‐stranded parallel β‐sheet. Recent studies suggest that the DD loop of TLR6 TIR domains and the BB loop of TIR module of TLR10 play critical roles in homotypic TLR interactions during TLR/IL‐1R signaling events.^38^, ^39^ This analysis propose that the TIR domain can form various types of dimers.

### 3.3 Interaction analysis for potential ligand‐ECD complexes formed by the gTLR21 protein

#### 3.3.1 Potential pockets analysis for LRR domains of gTLR21

**Figure 4:**
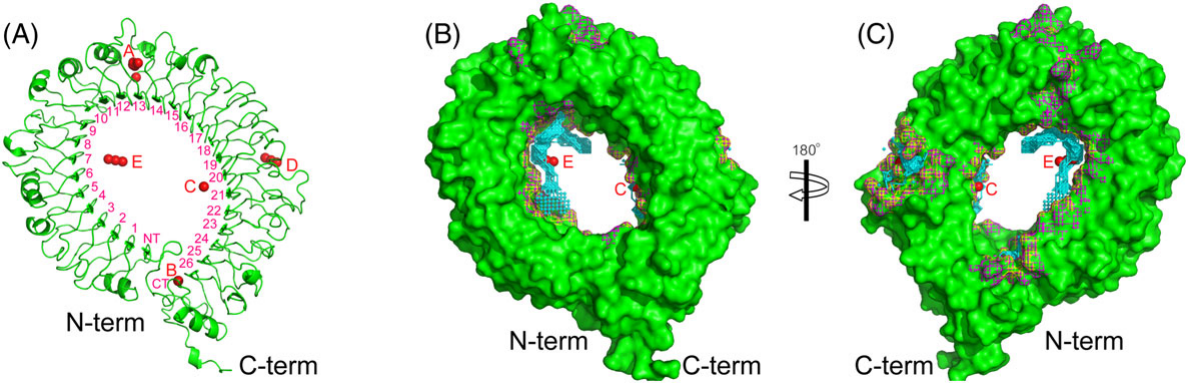
Mapping of predicted binding sites on the surface of the Gallus TLR21 (gTLR21) ectodomain (ECD). Five most likely binding sites are highlighted using red dots on the surface of the LRR model of gTLR21 by Meta Pocket2.0. The surfaces of the modeled structure of gTLR21 ECD are colored green, and the potential binding atoms and residues are indicated by yellow and magenta hatches, respectively. The potential binding clusters are indicated by cyan spheres

The Meta Pocket 2.0 server successfully predicted 5 most likely binding sites in the LRR domains of gTLR21 (Figure 4). The results of the prediction show that binding site A is a large hole in the center of the LRR domains (Figure 4A). A similar positioned pocket has previously been well studied in many TLRs. In the TLR1/TLR2 complex, this binding pocket of them not only is involved in ligand (lipopeptide) recognition but also is necessary for dimerization of TLRs 1 and 2.^40^ Protein‐protein interactions are also observed at a similar site in TLRs 7 and 8, which are required for homo‐dimerization of TLRs 7 and 8.^7^ These results reveal that the binding site A may be involved in the biological functions of gTLR21 ECD. Binding site E, which can form a relatively large surface pocket (Figure 4B), is also found in similar regions of the *Mus* TLR9 ECD and may play a role in recognizing the unmethylated CpG‐DNA motif.^8^ It is clear that a similar large hole in the inner concave face of gTLR21 ECD also constitutes a binding site C, which was previously identified in *Mus* TLR13 (Figure 4C). It has been proven that the binding site C is used for specific recognition of ssRNA.^9^ The remaining prediction results indicate the presence of small slit structures that may participate in binding other small potential ligands.

#### 3.3.2 Structural biology of the predicted CpG‐DNA interface within the ECD of gTLR21 complex

**Figure 5:**
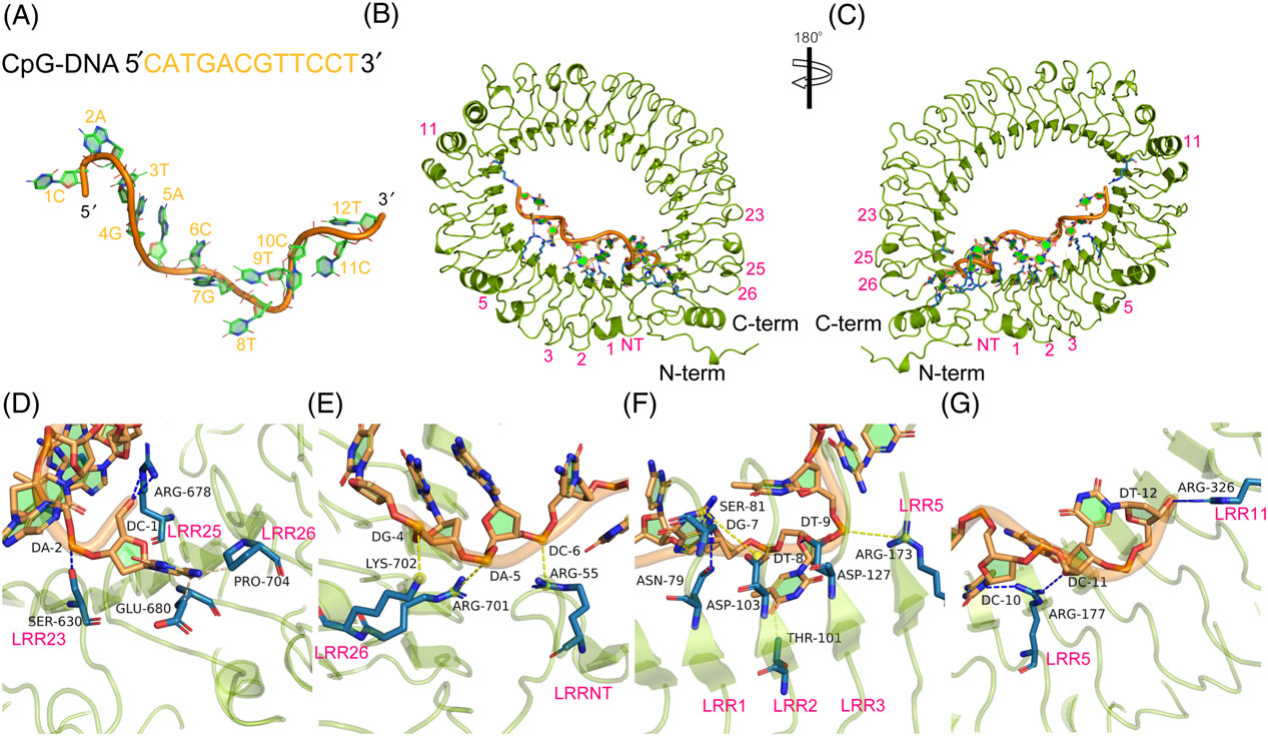
The sequence‐specific recognition mechanism of unmethylated cytosine phosphate guanine dideoxy nucleotide (CpG‐DNA) motif by Gallus TLR21 (gTLR21). A, The sequence and structure of CpG‐DNA. B, C, The unmethylated CpG‐DNA acts as a “molecular bridge” to penetrate through the concave face formed by the LRRNT domain, LRR1‐3, 5, and 11 of gTLR21. D, The base C1 in CpG‐DNA motif interfaces with Arg678, Glu680, and Pro704 via hydrogen bonds, and Ser630 in LRR23 forms a direct hydrogen bond with base A2. E, Potential salt bridges may form at the interface of Lys702‐G4, Arg701‐A5, and Arg55‐C6. F, A series of residues located in LRR1, LRR2, and LRR3 may also contribute to the maintenance of the structure of the gTLR21‐CpG‐DNA complex. G, The side chains of Arg177 of LRR5 and Arg326 of LRR11 are involved in interfacing with the bases C10‐T12 of 3 ′ arm of the CpG‐DNA. The side chains of gTLR21 are colored blue (in the ball‐and‐stick model), and the bases of the unmethylated CpG‐DNA motif are colored green, respectively. H‐bonds, hydrophobic interactions, and salt bridges are indicated by blue, yellow, and wheat dashed lines, respectively

The gTLR21 protein can recognize unmethylated CpG‐DNA as a “danger” ligand to alert the innate and adaptive immune systems; gTLR21 can activate downstream pathways affecting the immune system much like mammalian TLR9.^15^, ^16^ However, the mechanisms by which this specific recognition occurs have not yet been elaborated. Our results suggest that CpG‐DNA (Figure 5A) acts as a “molecular bridge” to penetrate through the concave face of gTLR21 LRR domains (Figure 5B). Our results on modeling structural features indicates that the interface comprising the LRRNT and LRRs 1‐3, 5, and 11 motifs can enhance the affinity of gTLR21 binding to CpG‐DNA (Figure 5C). Recent studies have also shown that the binding region spans from the LRRNT to LRR10 motif of TLR9.^8^

We also tried to identify the amino acid residues potentially involved in the interactions at the binding surface. Results show that the base C1 in CpG‐DNA motif forms direct hydrogen bonds with Arg678, Glu680, and Pro704 (Figure 5D). We have also found that Ser630 in LRR23 interfaces with the base A2 via hydrogen bond (Figure 5D). In the interaction domain, the bases G4, A5, and C6 potentially form salt bridges with Lys702, Arg701, and Arg55 (Figure 5E), respectively. Three amino acid residues are found to the interface with the G7 base via multiple intermolecular forces in which the Asn79 and Ser81 are located in LRR1 and the Asp103 located in LRR2. Thr101 of LRR2 and Asp127 of LRR3 are also devoting to interacting with T8 base. The backbone phosphates of T9 are recognized by Arg173 of LRR5 (Figure 5F). Simultaneously, the side chains of Arg177 and Arg326 conform an interface with the bases C10‐T12 of 3 ′ arm of the CpG‐DNA (Figure 5G). The structures of the agonistic CpG‐DNA bound to gTLR21 described in this study reveal the structural bases of CpG‐DNA recognition by gTLR21.

#### 3.3.3 Structural characteristics of the interaction of the potential ligand, ssRNA, with the LRR modules of gTLR21

**Figure 6:**
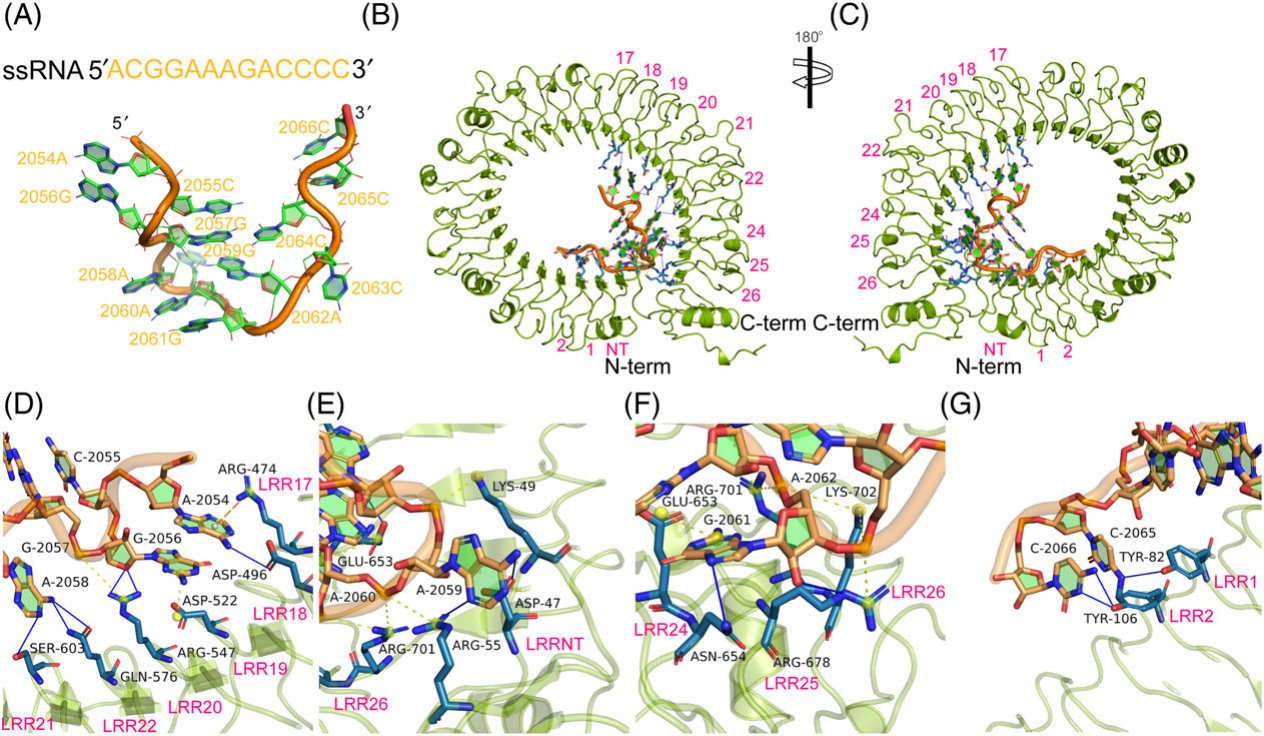
The sequence‐specific recognition mechanism of single‐stranded RNA (ssRNA) motif by Gallus TLR21 (gTLR21). A, The sequence and structure of ssRNA. B, C, The ssRNA could form a stem‐loop–like structure so that its 5 ′ and 3 ′ arms mainly fit along the inner concave surface formed by the C‐ and N‐terminal ends of the gTLR21 ECD, respectively. D, The bases A2054 and G2056‐2058 of the ssRNA can bind to the interaction surface surrounded by residues located in LRR17‐22 via potential π‐cation interaction, hydrogen bonds, and salt bridges. E, The backbone phosphates of base A2059 is recognized by Lys49 and form hydrogen bonds with Asp47 and Arg55, whereas the base A2060 interfaces with Arg55, Glu653, and Arg701 via salt bridges. F, The base G2061 in the ssRNA motif interfaces with Glu653‐Asn654 of LRR24, whereas Arg678 of LRR25 and Arg701 and Lys702 of LRR26 contribute to a combined interface with the base A2062 via multiple intermolecular forces. G, Tyr82 of LRR1 and Tyr106 of LRR2 are also involved in maintaining the structure of gTLR21‐ssRNA complex via hydrogen bonds. The side chains of gTLR21 are colored blue (in the ball‐and‐stick model), and the bases of ssRNA motif are green. H‐bonds, π‐ cation interactions, and salt bridges are indicated by blue, yellow, and wheat dashed lines, respectively

We have also investigated the potential binding mechanism of gTLR21 with ssRNA. The ssRNA molecule (Figure 6A) likely fits along the inner concave surface of gTLR21, with its 5 ′ and 3 ′ arms binding to the C‐ and N‐terminal ends of gTLR21 ECD (Figure 6B), respectively. This is consistent with how ssRNA molecules bind TLR13, except for opposite orientation of binding of TLR3–double‐stranded RNA complex.^6^ Structural features suggest that ssRNA also forms a stem‐loop–like structure that is highly similar to those observed in TLR13‐ssRNA complex (Figure 6C); the stem‐loop–like structure is also essential for TLR13 recognition of ssRNA.^9^, ^41^ Our structural biology analysis also strongly indicates that the predicted interaction surface is surrounded by the LRRNT, LRR1‐2, LRR17‐22, and LRR24‐26 motifs of the gTLR21 ECD to fix the ssRNA in position.

The amino acid residues, which could potentially participate in the interactions at the binding surface, are discussed here. Our results indicate that there may be a π‐cation interaction between the base A2054 and Arg474 of LRR17. The presence of hydrogen bonds is likely provided by the side chains of Asp496, Arg547, Gln576, and Ser603 in our results. Simultaneously, the bases G2056 and G2057 may potentially form salt bridges with Asp522 and Arg547, respectively, in the combined domain (Figure 6D). With the extension of the ssRNA motif, A2059 is recognized by Asp47, Arg55, and Lys49 via multiple intermolecular interactions. It is clear that the backbone phosphates of the base A2060 are recognized by Arg55, Glu653, and Arg701 (Figure 6E). The Glu653 toAsn654 of LRR24, Arg678 of LRR25, and Arg701 and Lys702 of LRR26 also contribute to the maintenance of this structure via different intermolecular forces (Figure 6F). The docking results indicate that Tyr82 of LRR1 and Tyr106 of LRR2 also devote to bind to the bases of 3 ′ arms of the ssRNA motif via hydrogen bonds (Figure 6G). Recently, a series of studies has demonstrated that *Mus* TLR13 is a receptor for the vesicular stomatitis virus^42^ and could detect sequence‐specifc areas of 23S ribosomal RNA from bacteria.^41^ The results of our study described here reveal that gTLR21 may have the potential to specifically recognize ssRNA like *Mus* TLR13^9^ and that this could contribute to the development of a strong immune mechanism in *Gallus*.

The TLRs ECD can form a horseshoe‐shaped structure with a concave surface that participates in the recognition of various pathogens. The signaling complex structures of TLRs with their respective ligands reveal diverse mechanisms in the recognition of a wide variety of PAMPs. These varied PAMPs have unique characteristic molecular signatures that are specially detected by the corresponding TLRs that recognize them. The results of LRR‐ligand complex prediction analysis indicate that gTLR21 may play an extensive role in mounting immune responses to bacteria and viruses via the recognition of specific nucleic acid ligands.

Because of selection pressures in the vertebrate evolutionary history of vertebrates, different classes of TLRs were generated to recognize the similar ligands and show analogous localization. Although TLR9 is absent in avian, fish genomes have both TLR9 and TLR21. Previous work has demonstrated that TLR9 and TLR21 have different ligand recognition profiles, and cooperatively mediate immune responses to CpG‐DNA in zebrafish.^17^ Recent studies have also proposed that the TLR21 gene is up‐expressed after infection by fish viruses in teleosts.^43^, ^44^ CpG‐DNA holds considerable promise as an adjuvant in vaccines target ssRNA viruses such as the Newcastle disease and influenza A virus H5N1.^45^, ^46^ These observations indicate that TLR21 may play an important role in mounting immune responses to the infections of specific pathogens that involve recognition of viral and bacterial RNA and DNA. These data indicate that TLR21 can play multiple roles in the innate immune system. Clearly, a detailed analysis of the structural characteristics of the gTLR21 ECD is needed to discriminate as other TLRs.

### 3.4 Homodimer analysis for the TIR domains of gTLR21

**Figure 7:**
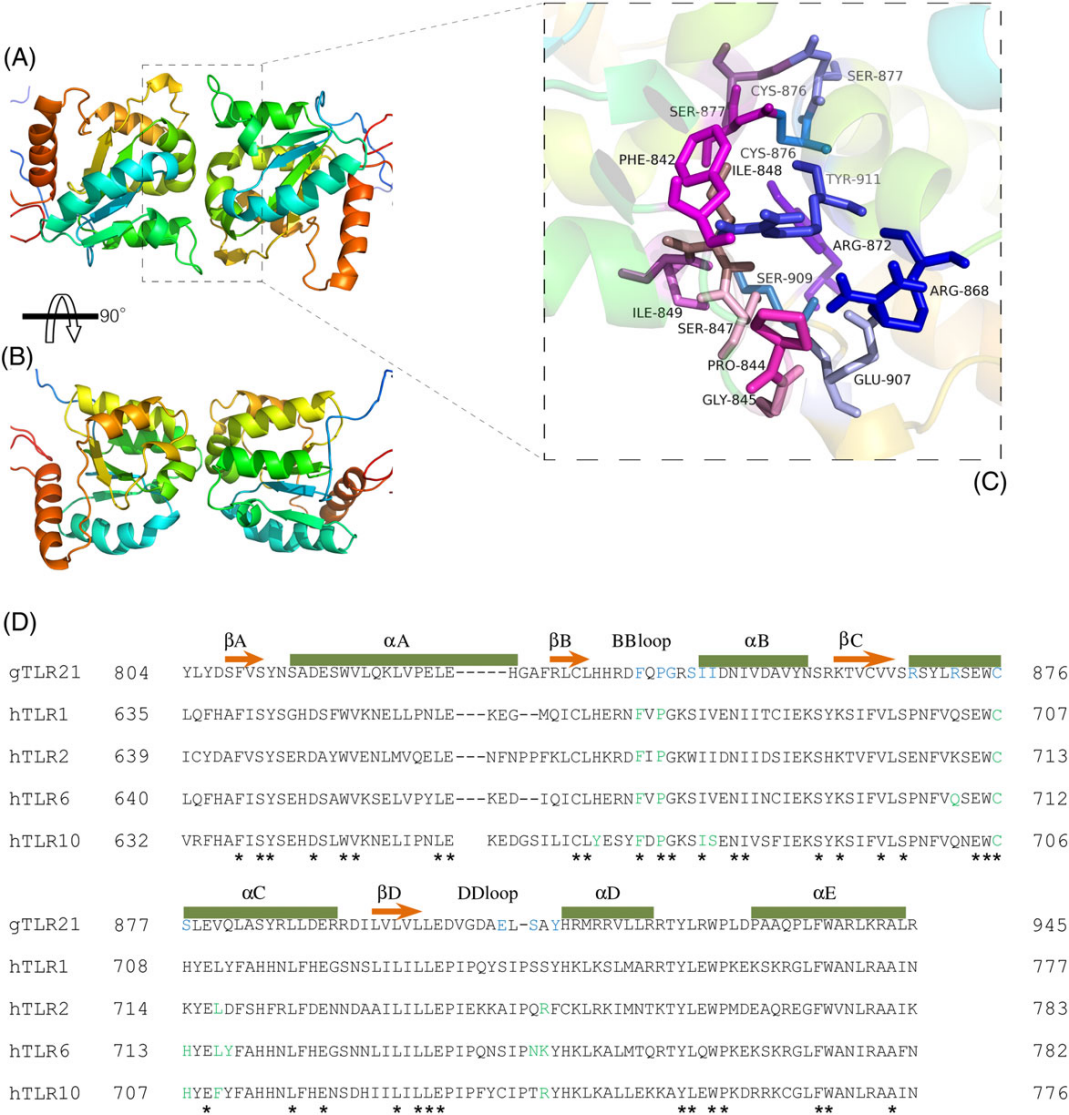
Homodimer of the Gallus TLR21 (gTLR21) intracellular (TIR) domain. A, The dimeric structure of the gTLR21 TIR domain shows the crystallographic asymmetric unit. B, Potential residues that could interact at the interface of each gTLR21 TIR domain monomer. C, Alignment of representative TIR domain sequences from different TLRs. The elements of secondary structures are indicated above each sequence. Amino acid sequences conserved across TLR families are marked by “*.” Previously identified amino acids that are known to be important for TLR function are colored green. The residues predicted to function in the interactions at the interface of gTLR21 are shown in blue

The docking results indicate that TIR monomers of gTLR21 are able to form homodimer complexes with one another (Figure 7A). Three‐dimensional structures of the gTLR21 TIR domains display a crystallographic asymmetric dimerism. Interaction analyses suggest that 12 amino acid residues (Phe842, Pro844, Gly845, Ser847, Ile848, Ile849, Arg868, Arg872, Cys876, Glu907, Ser909, and Tyr911) can contribute to the formation of the dimeric interface (Figure 7B). The results of the alignment analysis (Figure 7C) show that Phe842, Pro844, and Gly845, which located in the BB loop, are highly conserved in other TLRs and that residues in the DD loop (Glu907, Ser909, and Tyr911) have been reported to play an important role in the dimerization interface.^40^ We also find that Ile848 located in the αB helix and Cys876 located in the αC helix are highly conserved; both residues are likely to participate in forming TIR homodimers similar to those formed by TLR10.^39^

This study represents an attempt to apply computational methods such as protein‐protein docking analysis to explore the interactions of TIR domains to investigate the mechanisms of signaling induced by gTLR21. The homodimer analysis results suggest that the gTLR21 TIR region has the potential to form homodimers much like those formed by TLR6 and TLR10.^38^, ^39^ We have also found 3 potential amino acid residues (Phe842, Pro844, and Cys876) at the homodimer interface that highly conserved in other TLRs; these residues may play an essential role in signal transduction that triggered by TLR and interleukin‐1 (IL‐1).^38^, ^39^, ^47^ TLR9 was unable to elicit an immune response to unmethylated CpG‐DNA in MyD88 knockout mice,^48^ probably because the induction of type I interferons, particularly type I interferon‐α, by TLR9 depends on the MyD88‐IRF7 pathway in pDC cells.^49^ TLR13 also appears to induce a MyD88‐dependent signaling pathway to trigger the activation of NF‐κB; and TLR13 is also dependent on IRF7 for activating type 1 interferon pathways.^42^ Results of recent studies indicate that gTLR21 ectopic expressed in HEK‐293 cells could regulate NF‐κB and furthermore could mediate the expression of cytokines in HD11 cells on both exogenous CpG‐DNA stimulation.^15^, ^16^ Taken together, these data indicate that the TIR domain participates in protein‐protein interactions with intracellular adaptor proteins for signaling processes.

## 4 CONCLUSIONS

By performing a phylogenetic and evolutionary analysis of TLR21 proteins from majority animals, we report that TLR21 is phylogenetically related to TLR11 family and is perhaps a close ortholog of *Mus* TLR13. Our structural biology analysis suggests that there is no Z‐loop in the gTLR21 ECD, although the Z‐loop is known to play a critical role in activation for the TLR7 family. The molecular structural features of the gTLR21 TIR domain indicate that it contains a central 4‐stranded parallel β‐sheet surrounded by 5 α‐helices on both sides. The TIR domains of gTLR21 are also highly conserved, and similar to other TLRs. Molecular docking analysis has revealed that gTLR21 has the potential to distinguish between different ligands. The results of the homodimer analysis suggest that the TIR domain may be involved in forming gTLR21 homodimers. The Cys876 from the αC helix as well as the Phe842 and Pro844 from the BB loop are identical in the TIR domains of other TLRs and may function as docking sites in TIR domain homodimers.

## CONFLICT OF INTEREST

The authors declare to have no conflict of interest.

## FUNDING

This work was financially supported by National Natural Science Foundation of China (31572389).

## Supporting information


**Figure S1.** Ramachandran plot analysis of the models developed for the LRR motif (A) and the TIR domain (B) of Gallus TLR21 (gTLR21) to check the stereochemical quality of a target protein model structure by analyzing residue‐by‐residue geometry and overall structure geometry. The final target model of the ECD and TIR domains of gTLR21 have >98% and >99.4% of their respective residue j‐ψ angles in the allowed regions of Ramachandran plot.
**Figure S2.** Verify3D curves of the models developed for the LRR motif (A) and the TIR domain (B) of Gallus TLR21 (gTLR21). This score measures the compatibility of a generated model with its sequence by using a scoring function. (A) As ~93.14% of the residues have a compatibility score of above 0.2 in the Verify3D graph, this suggests that the model of gTLR21 ECD is self‐consistent in terms of structure compatibility. (B) About 86.29% of the residues compatibility score of above 0.2 in the Verify3D graph for the model of the TIR domain of gTLR21. Residues located in the C‐terminal are far from the substrate binding domain; these results suggest that the model of gTLR21 TIR domain is largely self‐consistent in terms of structure compatibility.
**Figure S3.** Curves statistics of non‐bonded interactions between different atom types and plots for the models developed for the LRR motif (A) and the TIR domain (B) of Gallus TLR21 (gTLR21) as calculated by ERRAT2. The overall quality factors of the models for the LRR and TIR domain of gTLR21 are >90 and >97 points, respectively, and indicate the the total mass factor of non‐bonded atom interactions is reasonable. “*” On the error axis, two line are drawn to indicate the confidence with which it is possible to reject regions that exceed that error value. “**” Expressed as the percentage of the protein for which the calculated error value falls below the follow the 95% rejection limit. Good high resolution structure generally produce values around 95% or higher. For lower resolutions (2.5 to 3 Å) the average overall quality factor is around 91%.
**Figure S4.** ProSA‐web display scores and energy plots of the models developed for the LRR motif (A) and TIR domain (B) of Gallus TLR21 (gTLR21) using knowledge‐based energy profiles. (A) The model of gTLR21 ECD has a score of ‐6.02 in ProSA‐web, indicating that the LRR motif model of gTLR21 is well within the range of a typical native structure. (B) The model of gTLR21 TIR domain has a score of ‐4.07, which is similar to ‐4.28, the score obtained by molecule 2j67 in ProSA‐web; this indicates that the model of g TLR21 TIR domain is well within the range of a typical native structure.
