# Supplementary figures and images for: The evolutionary characteristics and structural biology of Gallus toll‐like receptor 21
Source: J Mol Recognit. 2017 Dec 27;31(6):e2696. doi: 10.1002/jmr.2696 (PMC6001672; doi:10.1002/jmr.2696)

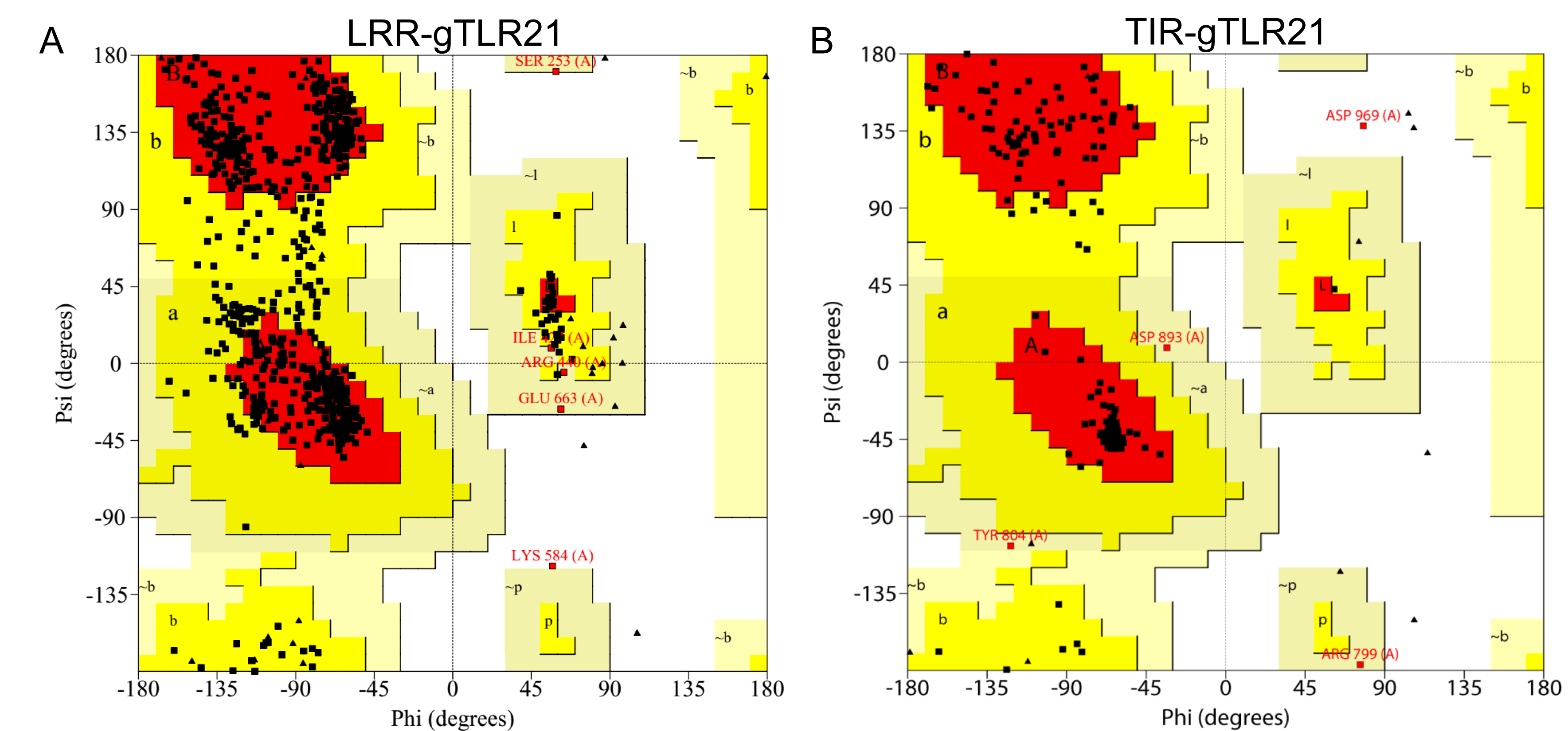

Supplement: Supplementary file 1 — Figure S1. Ramachandran plot analysis of the models developed for the LRR motif (A) and the TIR domain (B) of Gallus TLR21 (gTLR21) to check the stereochemical quality of a target protein model structure by analyzing residue‐by‐residue geometry and overall structure geometry. The final target model of the ECD and TIR domains of gTLR21 have >98% and >99.4% of their respective residue j‐ψ angles in the allowed regions of Ramachandran plot. Figure S2. Verify3D curves of the models developed for the LRR motif (A) and the TIR domain (B) of Gallus TLR21 (gTLR21). This score measures the compatibility of a generated model with its sequence by using a scoring function. (A) As ~93.14% of the residues have a compatibility score of above 0.2 in the Verify3D graph, this suggests that the model of gTLR21 ECD is self‐consistent in terms of structure compatibility. (B) About 86.29% of the residues compatibility score of above 0.2 in the Verify3D graph for the model of the TIR domain of gTLR21. Residues located in the C‐terminal are far from the substrate binding domain; these results suggest that the model of gTLR21 TIR domain is largely self‐consistent in terms of structure compatibility. Figure S3. Curves statistics of non‐bonded interactions between different atom types and plots for the models developed for the LRR motif (A) and the TIR domain (B) of Gallus TLR21 (gTLR21) as calculated by ERRAT2. The overall quality factors of the models for the LRR and TIR domain of gTLR21 are >90 and >97 points, respectively, and indicate the the total mass factor of non‐bonded atom interactions is reasonable. “*” On the error axis, two line are drawn to indicate the confidence with which it is possible to reject regions that exceed that error value. “**” Expressed as the percentage of the protein for which the calculated error value falls below the follow the 95% rejection limit. Good high resolution structure generally produce values around 95% or higher. For lower resolutions (2.5 to 3 Å) [file JMR-31-na-s001.zip › Fig-S1-Ramachandran Plot.tif]

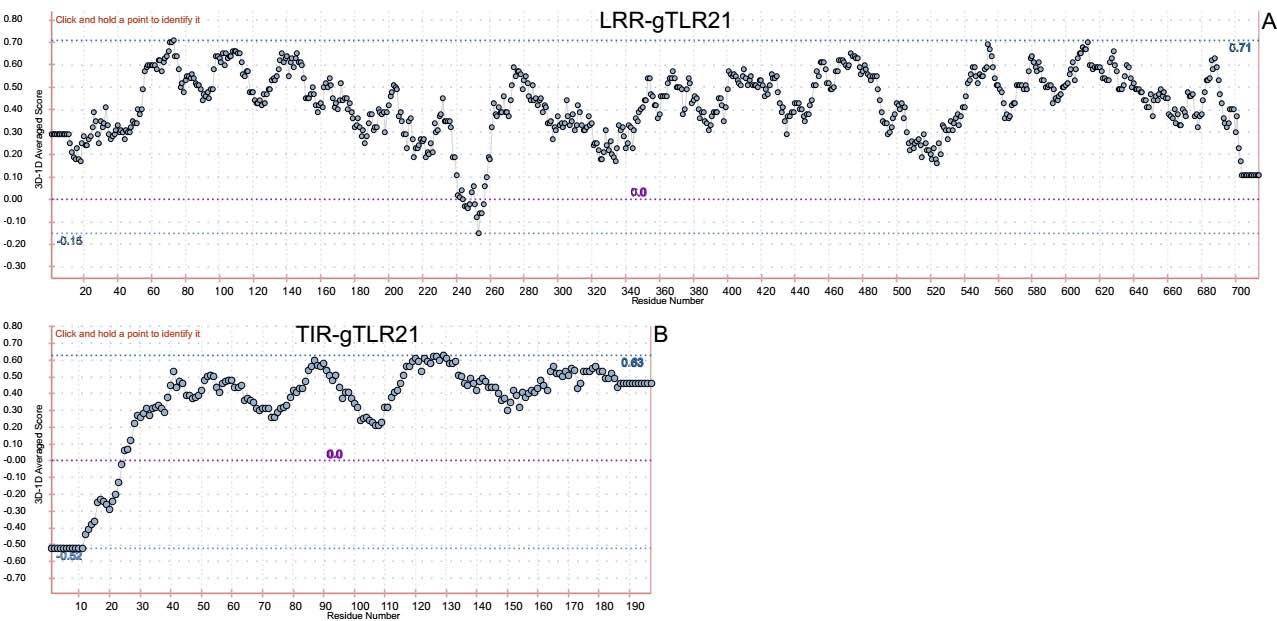

Supplement: Supplementary file 1 — Figure S1. Ramachandran plot analysis of the models developed for the LRR motif (A) and the TIR domain (B) of Gallus TLR21 (gTLR21) to check the stereochemical quality of a target protein model structure by analyzing residue‐by‐residue geometry and overall structure geometry. The final target model of the ECD and TIR domains of gTLR21 have >98% and >99.4% of their respective residue j‐ψ angles in the allowed regions of Ramachandran plot. Figure S2. Verify3D curves of the models developed for the LRR motif (A) and the TIR domain (B) of Gallus TLR21 (gTLR21). This score measures the compatibility of a generated model with its sequence by using a scoring function. (A) As ~93.14% of the residues have a compatibility score of above 0.2 in the Verify3D graph, this suggests that the model of gTLR21 ECD is self‐consistent in terms of structure compatibility. (B) About 86.29% of the residues compatibility score of above 0.2 in the Verify3D graph for the model of the TIR domain of gTLR21. Residues located in the C‐terminal are far from the substrate binding domain; these results suggest that the model of gTLR21 TIR domain is largely self‐consistent in terms of structure compatibility. Figure S3. Curves statistics of non‐bonded interactions between different atom types and plots for the models developed for the LRR motif (A) and the TIR domain (B) of Gallus TLR21 (gTLR21) as calculated by ERRAT2. The overall quality factors of the models for the LRR and TIR domain of gTLR21 are >90 and >97 points, respectively, and indicate the the total mass factor of non‐bonded atom interactions is reasonable. “*” On the error axis, two line are drawn to indicate the confidence with which it is possible to reject regions that exceed that error value. “**” Expressed as the percentage of the protein for which the calculated error value falls below the follow the 95% rejection limit. Good high resolution structure generally produce values around 95% or higher. For lower resolutions (2.5 to 3 Å) [file JMR-31-na-s001.zip › Fig-S2-Verify3d.tif]

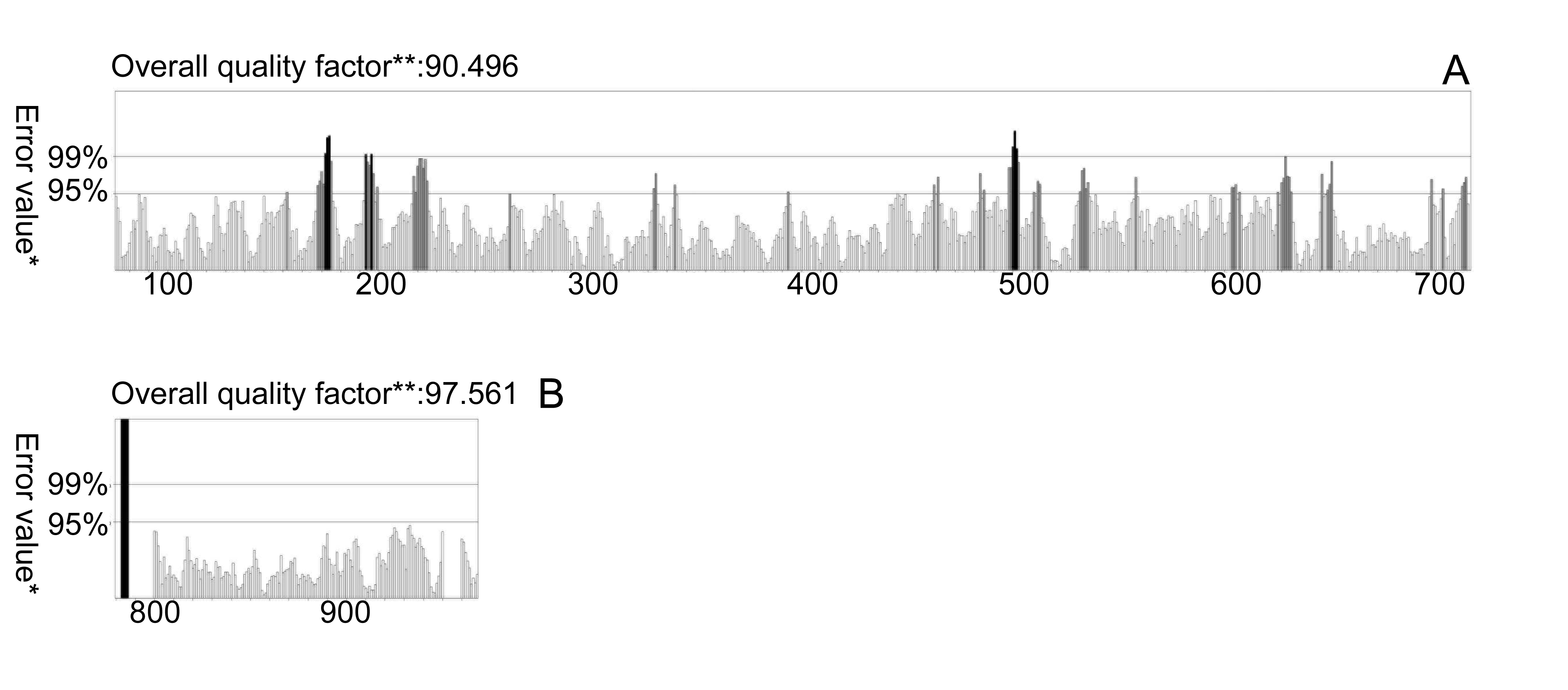

Supplement: Supplementary file 1 — Figure S1. Ramachandran plot analysis of the models developed for the LRR motif (A) and the TIR domain (B) of Gallus TLR21 (gTLR21) to check the stereochemical quality of a target protein model structure by analyzing residue‐by‐residue geometry and overall structure geometry. The final target model of the ECD and TIR domains of gTLR21 have >98% and >99.4% of their respective residue j‐ψ angles in the allowed regions of Ramachandran plot. Figure S2. Verify3D curves of the models developed for the LRR motif (A) and the TIR domain (B) of Gallus TLR21 (gTLR21). This score measures the compatibility of a generated model with its sequence by using a scoring function. (A) As ~93.14% of the residues have a compatibility score of above 0.2 in the Verify3D graph, this suggests that the model of gTLR21 ECD is self‐consistent in terms of structure compatibility. (B) About 86.29% of the residues compatibility score of above 0.2 in the Verify3D graph for the model of the TIR domain of gTLR21. Residues located in the C‐terminal are far from the substrate binding domain; these results suggest that the model of gTLR21 TIR domain is largely self‐consistent in terms of structure compatibility. Figure S3. Curves statistics of non‐bonded interactions between different atom types and plots for the models developed for the LRR motif (A) and the TIR domain (B) of Gallus TLR21 (gTLR21) as calculated by ERRAT2. The overall quality factors of the models for the LRR and TIR domain of gTLR21 are >90 and >97 points, respectively, and indicate the the total mass factor of non‐bonded atom interactions is reasonable. “*” On the error axis, two line are drawn to indicate the confidence with which it is possible to reject regions that exceed that error value. “**” Expressed as the percentage of the protein for which the calculated error value falls below the follow the 95% rejection limit. Good high resolution structure generally produce values around 95% or higher. For lower resolutions (2.5 to 3 Å) [file JMR-31-na-s001.zip › Fig-S3-ERRAT.tif]

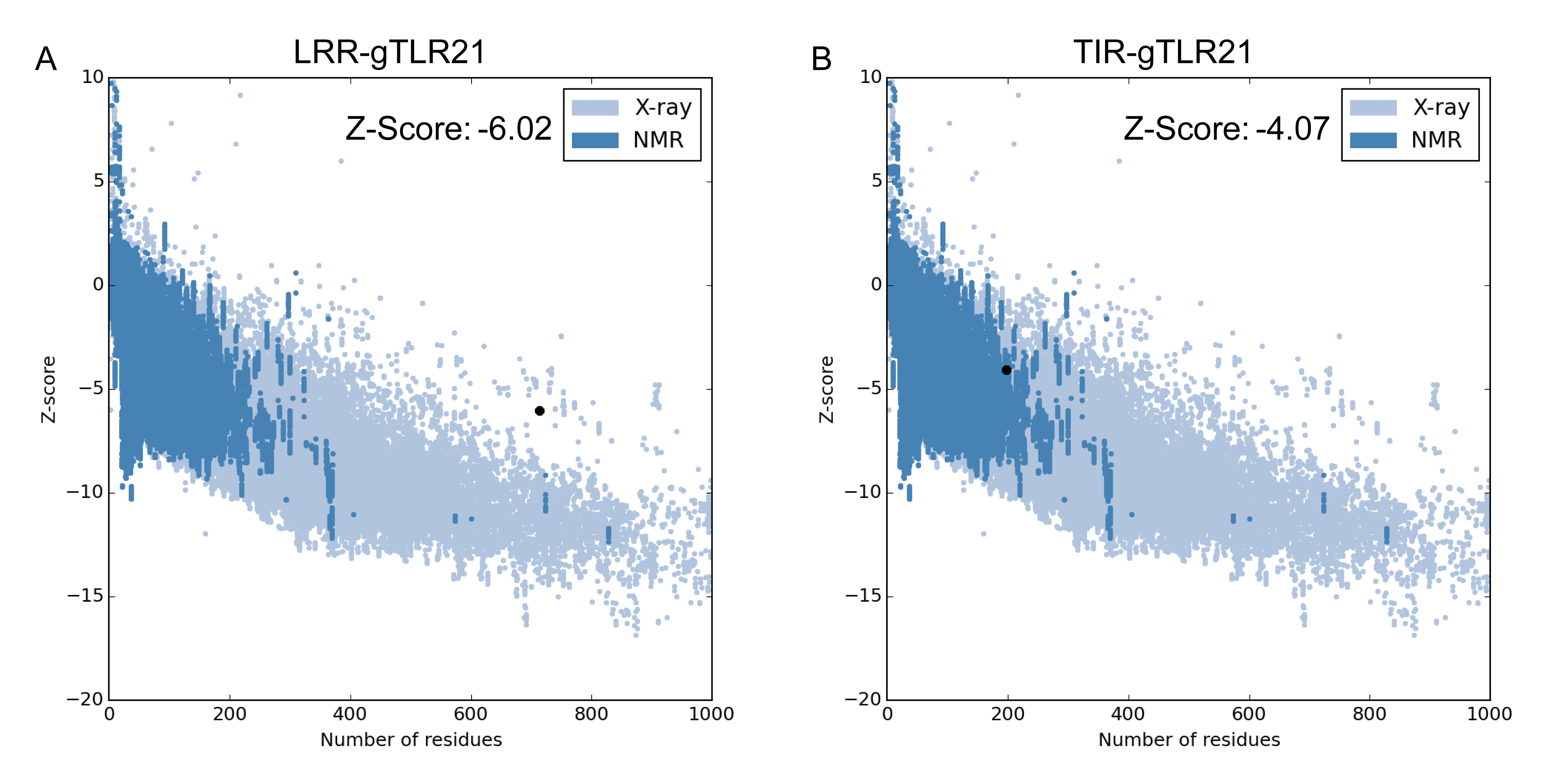

Supplement: Supplementary file 1 — Figure S1. Ramachandran plot analysis of the models developed for the LRR motif (A) and the TIR domain (B) of Gallus TLR21 (gTLR21) to check the stereochemical quality of a target protein model structure by analyzing residue‐by‐residue geometry and overall structure geometry. The final target model of the ECD and TIR domains of gTLR21 have >98% and >99.4% of their respective residue j‐ψ angles in the allowed regions of Ramachandran plot. Figure S2. Verify3D curves of the models developed for the LRR motif (A) and the TIR domain (B) of Gallus TLR21 (gTLR21). This score measures the compatibility of a generated model with its sequence by using a scoring function. (A) As ~93.14% of the residues have a compatibility score of above 0.2 in the Verify3D graph, this suggests that the model of gTLR21 ECD is self‐consistent in terms of structure compatibility. (B) About 86.29% of the residues compatibility score of above 0.2 in the Verify3D graph for the model of the TIR domain of gTLR21. Residues located in the C‐terminal are far from the substrate binding domain; these results suggest that the model of gTLR21 TIR domain is largely self‐consistent in terms of structure compatibility. Figure S3. Curves statistics of non‐bonded interactions between different atom types and plots for the models developed for the LRR motif (A) and the TIR domain (B) of Gallus TLR21 (gTLR21) as calculated by ERRAT2. The overall quality factors of the models for the LRR and TIR domain of gTLR21 are >90 and >97 points, respectively, and indicate the the total mass factor of non‐bonded atom interactions is reasonable. “*” On the error axis, two line are drawn to indicate the confidence with which it is possible to reject regions that exceed that error value. “**” Expressed as the percentage of the protein for which the calculated error value falls below the follow the 95% rejection limit. Good high resolution structure generally produce values around 95% or higher. For lower resolutions (2.5 to 3 Å) [file JMR-31-na-s001.zip › Fig-S4-ProSA.tif]
